# Optimized preoperative planning of double outlet right ventricle patients by 3D printing and virtual reality: a pilot study

**DOI:** 10.1093/icvts/ivad072

**Published:** 2023-05-18

**Authors:** Jette J Peek, Wouter Bakhuis, Amir H Sadeghi, Kevin M Veen, Arno A W Roest, Nico Bruining, Theo van Walsum, Mark G Hazekamp, Ad J J C Bogers

**Affiliations:** Department of Cardiothoracic Surgery, Erasmus MC, University Medical Center Rotterdam, Thoraxcenter, Rotterdam, Netherlands; Department of Cardiothoracic Surgery, Erasmus MC, University Medical Center Rotterdam, Thoraxcenter, Rotterdam, Netherlands; Department of Cardiothoracic Surgery, Erasmus MC, University Medical Center Rotterdam, Thoraxcenter, Rotterdam, Netherlands; Department of Cardiothoracic Surgery, Erasmus MC, University Medical Center Rotterdam, Thoraxcenter, Rotterdam, Netherlands; Department of Pediatric Cardiology, Leiden University Medical Center, Leiden, Netherlands; Department of Clinical Epidemiology and Innovation (KEI), Erasmus MC, University Medical Center Rotterdam, Rotterdam, Netherlands; Department of Radiology & Nuclear Medicine, Erasmus MC, University Medical Center Rotterdam, Rotterdam, Netherlands; Department of Cardiothoracic Surgery, Leiden University Medical Center, Leiden, Netherlands; Department of Cardiothoracic Surgery, Erasmus MC, University Medical Center Rotterdam, Thoraxcenter, Rotterdam, Netherlands

**Keywords:** Virtual reality, 3D printing, Surgical planning, Double outlet right ventricle, Congenital heart disease, Congenital cardiac surgery

## Abstract

**OBJECTIVES:**

In complex double outlet right ventricle (DORV) patients, the optimal surgical approach may be difficult to assess based on conventional 2-dimensional (2D) ultrasound (US) and computed tomography (CT) imaging. The aim of this study is to assess the added value of 3-dimensional (3D) printed and 3D virtual reality (3D-VR) models of the heart used for surgical planning in DORV patients, supplementary to the gold standard 2D imaging modalities.

**METHODS:**

Five patients with different DORV subtypes and high-quality CT scans were selected retrospectively. 3D prints and 3D-VR models were created. Twelve congenital cardiac surgeons and paediatric cardiologists, from 3 different hospitals, were shown 2D-CT first, after which they assessed the 3D print and 3D-VR models in random order. After each imaging method, a questionnaire was filled in on the visibility of essential structures and the surgical plan.

**RESULTS:**

Spatial relationships were generally better visualized using 3D methods (3D printing/3D-VR) than in 2D. The feasibility of ventricular septum defect patch closure could be determined best using 3D-VR reconstructions (3D-VR 92%, 3D print 66% and US/CT 46%, *P* < 0.01). The percentage of proposed surgical plans corresponding to the performed surgical approach was 66% for plans based on US/CT, 78% for plans based on 3D printing and 80% for plans based on 3D-VR visualization.

**CONCLUSIONS:**

This study shows that both 3D printing and 3D-VR have additional value for cardiac surgeons and cardiologists over 2D imaging, because of better visualization of spatial relationships. As a result, the proposed surgical plans based on the 3D visualizations matched the actual performed surgery to a greater extent.

**Figure:**
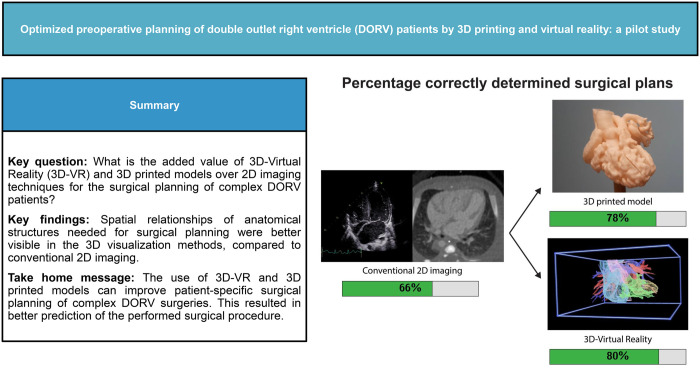

## INTRODUCTION

Double outlet right ventricle (DORV) is a complex congenital heart disease (CHD), in which both the pulmonary artery and the aorta originate predominantly (>50%) or completely from the morphologically right ventricle. Of all patients with CHD, DORV has an incidence of 1.0–1.5%, and it is diagnosed in 1/10 000 live births [1]. For surgical planning of each individual DORV patient, thorough and accurate evaluation of the intracardiac anatomy and relationships is required to determine whether biventricular repair or univentricular palliation is the best possible approach. Eleven important anatomical structures, called *essential modifiers*, are relevant for the surgical planning and clinical outcomes of DORV [2]. An adequate surgical plan based on this anatomical information is of utmost importance; it increases precision, minimizes the amount of unexpected findings, avoids intraoperative improvisation, decreases intraoperative time and associated comorbidities, and therefore results in better outcomes [3–5]. However, accurate preoperative planning is difficult, since the surgical approach is different for each individual in this heterogenous patient group [6]. Moreover, often these patients require multiple surgical procedures, increasing the complexity even more by scar tissue, fibrosis and adhesions during reoperations [7, 8].

**Figure 1:**
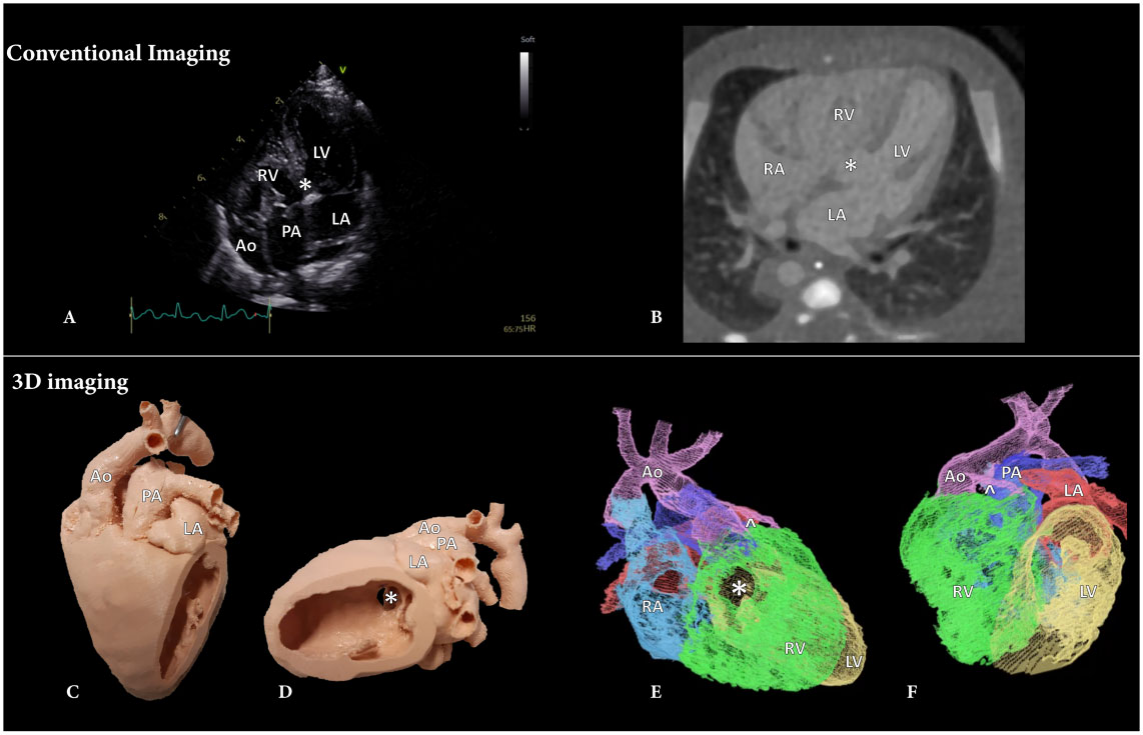
Imaging modalities used in double outlet right ventricle patients. (**A**) Ultrasound 4-chamber overview, (**B**) axial CT image, (**C**) anterior view of the 3D printed model, (**D**) lateral view of the 3D printed model, (**E**) right lateral view of the 3D-VR reconstruction, (**F**) anterior view of the 3D-VR reconstruction. Ao: aorta; LA: left atrium; LV: left ventricle; PA: pulmonary artery; RA: right atrium; RV: right ventricle; ^: coronary artery; *: ventricular septum defect.

Currently, diagnosis and preoperative surgical planning of DORV is achieved based on ultrasound (US) images (Fig. 1A), which in some cases is insufficient to visualize the complex anatomy and spatial intracardiac relationships of the heart [9]. In these cases, cross-sectional contrast enhanced computed tomography (CT) images (Fig. 1B), Magnetic Resonance Imaging can be acquired [10]. These modalities are generally visualized on a 2-dimensional (2D) screen and need to be mentally translated to a 3-dimensional (3D) volume to understand the anatomy [9]. For this mental 3D reconstruction, experience and advanced knowledge of the anatomy and 3D spatial orientation is required, especially with the abnormal anatomy in DORV patients [7, 11, 12].

Several 3D visualization technologies have been suggested to facilitate surgical planning, including 3D printing (Fig. 1C and D) and 3D virtual reality (3D-VR) (Fig. 1E and F). The applications of 3D printing and 3D-VR reconstructions are already being explored; however, studies comparing these different 3D visualizations are still scarce, and use of 3D is currently not implemented as standard surgical planning in CHD surgery [13, 14]. The aim of this study is to assess the added value of these 3D visualization methods (3D-VR models and 3D printed models) for surgical planning in DORV patients, supplementary to the gold standard 2D imaging modalities, in a retrospective clinical setting.

## PATIENTS AND METHODS

### Ethics statement

The Erasmus MC Medical Ethical Review Committee approved the research protocol (MEC-2020–0891). Participants provided verbal informed consent before participating.

### 3D model reconstructions

**Figure 2:**
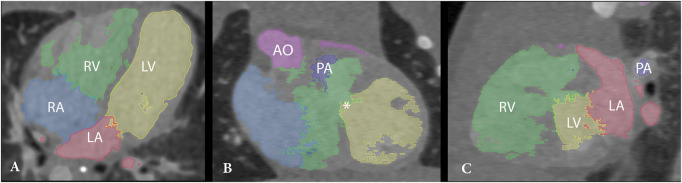
CT scan with segmentations of the cardiac structures. An axial (**A**), coronal (**B**) and sagittal (**C**) cross-sectional slice are shown, together with the highlighted cardiac segments. Ao: aorta; LA: left atrium; LV: left ventricle; PA: pulmonary artery; RA: right atrium; RV: right ventricle; ^: coronary artery; *: ventricular septum defect.

Operated DORV patients were selected retrospectively for 3D reconstruction if the decision of the surgical strategy was difficult based on anatomical complexity. In order to reconstruct the 3D printed and 3D-VR models, the CT images were collected, anonymized and exported as DICOM files (Digital Imaging and Communications in Medicine) from the Picture Archiving and Communication System. The 3D printed models were fabricated according to a previously published protocol [15]. To create 3D-VR reconstructions, segmentation of cardiac structures was performed semiautomatically by using grey value thresholding, region growing algorithms, and manual editing using 3D Slicer software [16] (Fig. 2). Segmentations were exported as binary NifTi masks and were directly loaded in combination with the DICOM CT images in 3D-VR visualization software using a MedicalVR workstation and software (MedicalVR, Amsterdam, The Netherlands).

### Study participants and study setup

**Figure 3:**
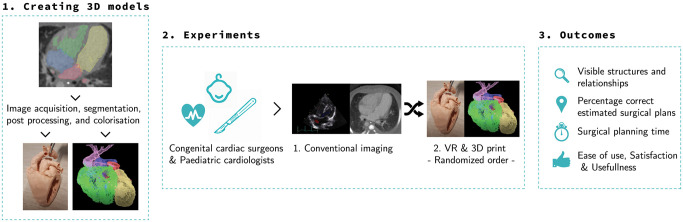
Overview of the workflow; creating 3D models, performing experiments and outcomes.

All participants were congenital cardiothoracic surgeons and paediatric cardiologists. Participants were blinded for the executed surgical procedure of the patients. An overview of the hereafter described workflow and outcomes is shown in Fig. 3. The participants were shown all images and 3D models of the selected patients. For each retrospective patient case, the participants were first shown the gold standard imaging (diagnostic preoperative US and CT images). Subsequently, the participant assessed the 3D visualization methods (3D printed and 3D-VR models) of the corresponding patient (Video 1). The order of assessment of the 2 3D visualization methods was random, to overcome sequence bias. Right after assessing each image visualization method, the participants filled in a questionnaire based on the visibility of the essential modifiers (3-point Likert-scale: poor, neutral, good) and proposed a surgical plan. This surgical plan was then compared in retrospect with the actually performed surgery. Moreover, participants rated their certainty on the proposed surgical plan on a scale from 0 (completely unsure) to 10 (100% sure) (Supplementary Material, File SA). Above mentioned steps were performed for all 5 patient cases. When a participant had finalized all patient cases, a questionnaire was filled in on the usefulness, satisfaction and ease of use [17]. Advantages and disadvantages were filled out for both 3D printing and 3D-VR reconstruction (Supplementary Material, File SB).

### Statistical analysis

Statistical analysis was performed using R (R Core Team, Vienna, Austria, www.R-project.org). Because the 12 participants assessed 5 imaging datasets of complex DORV patients, clustering could exist within data filled in by one participant and also within the data of one patient. Therefore, for the essential modifiers, a linear mixed-effect model with random intercept for patients and participants was used to account for clustering. A null-model was compared to a model containing the different imaging methods as covariates using a likelihood ratio test based on Galbraith *et al.* [18]. Data were divided into 3 groups (CT/US, VR, 3D printing) with different clusters (participants and patients). First, a null-model was made, in which only the patient and participant clustering was taken into account. This was done by specifying a random intercept for patient and participant (crossed random effects). Moreover, a second model was made, including also the 3 imaging methods as fixed effects, besides the clustering of patients and participants. These 2 models were compared using a likelihood-ratio test, in order to calculate the *P* value. Next, to assess between which 2 groups the results are statistically significantly different, a *post hoc* analysis was performed using these models and a clustered Wilcoxon signed rank test from the package ‘clusrank’ [19]. Moreover, Bonferroni correction was performed to correct for multiple testing, such that a *P* value < 0.00119 was considered statistically significant. Continuous data are presented as median with interquartile range (IQR) and categorical data are presented as percentage (frequency).

## RESULTS

**Figure 4:**
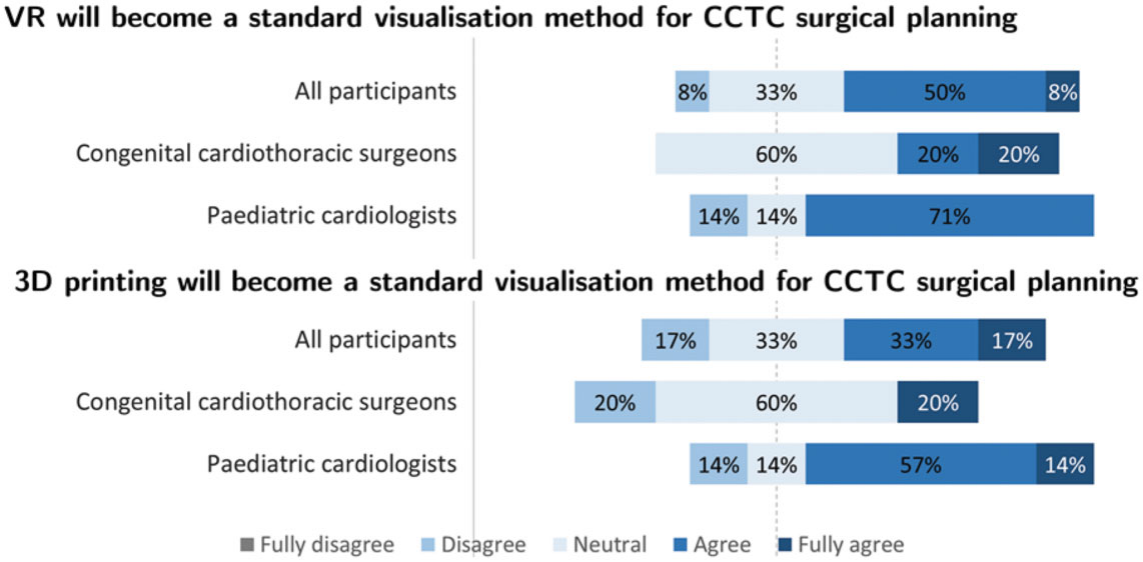
Participants’ opinion towards future use of 3D visualization.

Twelve participants, 5 congenital cardiothoracic surgeons and 7 paediatric cardiologists, working in 3 different medical centres, were included in this study (Table 1). The participants had a median work experience of 16 years (IQR: 13–25). Eleven complex DORV patients with preoperatively available 3D printed models were identified. Five representative patient cases were selected, since these patients had various anatomical DORV variants, underwent different repair surgeries and sufficient CT scan quality for 3D-VR reconstructions (contrast enhanced CT with a maximum slice thickness of 0.6 mm). The segmentations of the cardiac structures took approximately 60–120 min per patient, depending on anatomical complexity. All 5 patients underwent successful surgery, the patient characteristics are shown in Table 2 and the opinion towards future use of the 3D methods in Fig. 4. Nine of the participants assessed all 5 patient cases, the other participants assessed 1–3 patient cases.

**Table 1:** Participant characteristics

	All participants (*n* = 12)	Congenital cardiothoracic surgeons (*n* = 5)	Paediatric cardiologists (*n* = 7)
Work experience (years), median (IQR)			
	16.0 (13–25)	17.5 (8–28)	16.0 (14–24)
Gaming experience, % (*n*)			
Never	25 (3)	0 (0)	43 (3)
Few times	75 (9)	100 (5)	57 (4)
Regular basis	0 (0)	0 (0)	0 (0)
Attitude towards new technologies, % (*n*)			
Innovators	0 (0)	0 (0)	0 (0)
Early adopters	42 (5)	60 (3)	29 (2)
Early majority	25 (3)	0 (0)	43 (3)
Late majority	33 (4)	40 (2)	29 (2)
Laggards	0 (0)	0 (0)	0 (0)
Virtual reality experience, % (*n*)			
Never	8 (1)	0 (0)	14 (1)
Few times	92 (11)	100 (5)	86 (6)
Regular basis	0 (0)	0 (0)	0 (0)
Expert	0 (0)	0 (0)	0 (0)
3D print experience, % (*n*)			
Never	0 (0)	0 (0)	0 (0)
Few times	75 (9)	80 (4)	71 (5)
Regular basis	25 (3)	20 (1)	29 (2)

Based on self-completed questionnaires, the following characteristics of participants were collected. Inconsistencies in the sum of percentages is due to the rounding of the percentages.

IQR: interquartile range.

**Table 2:** Patient characteristics

Patient	Type DORV	Associated anomalies	Surgical approach	Performed procedure	Previous palliative surgeries	Age at surgery (days)
1	TOF type	ASD type 2, right AA, hypoplastic PA, single coronary ostium, double VCS, azygos continuation	Biventricular	Intraventricular tunnel, RVOT enlargement with TAP	Central shunt	254
2	ncVSD type	ASD type 2, CoA	Biventricular	Intraventricular tunnel	PA banding, coarctectomy	600
3	TGA type	LVOTO, ASD type 2	Biventricular	Nikaidoh procedure	MBTS	319
4	ccTGA type	PS, single coronary ostium, Ebstein TV	Univentricular	Fontan procedure	Bidirectional Glenn, ASD creation	2468
5	TGA type	Subpulmonary accessory tissue without obstruction	Biventricular	Intraventricular tunnel, arterial switch		84

AA: aortic arch; ASD: atrial septum defect; ccTGA: congenitally corrected TGA; CoA: coarctation aortae; LVOTO: left ventricular outflow tract obstruction; MBTS: modified Blalock–Taussig Shunt; ncVSD: non-committed ventricular septum defect; PA: pulmonary artery; PS: pulmonary valve stenosis; PFO: persistent foramen ovale; RVOT: right ventricular outflow tract; TAP: transannular patch; TGA: transposition of the great arteries; TOF: Tetralogy of Fallot; TV: tricuspid valve; VCS: superior vena cava.

### Essential modifiers

**Figure 5:**
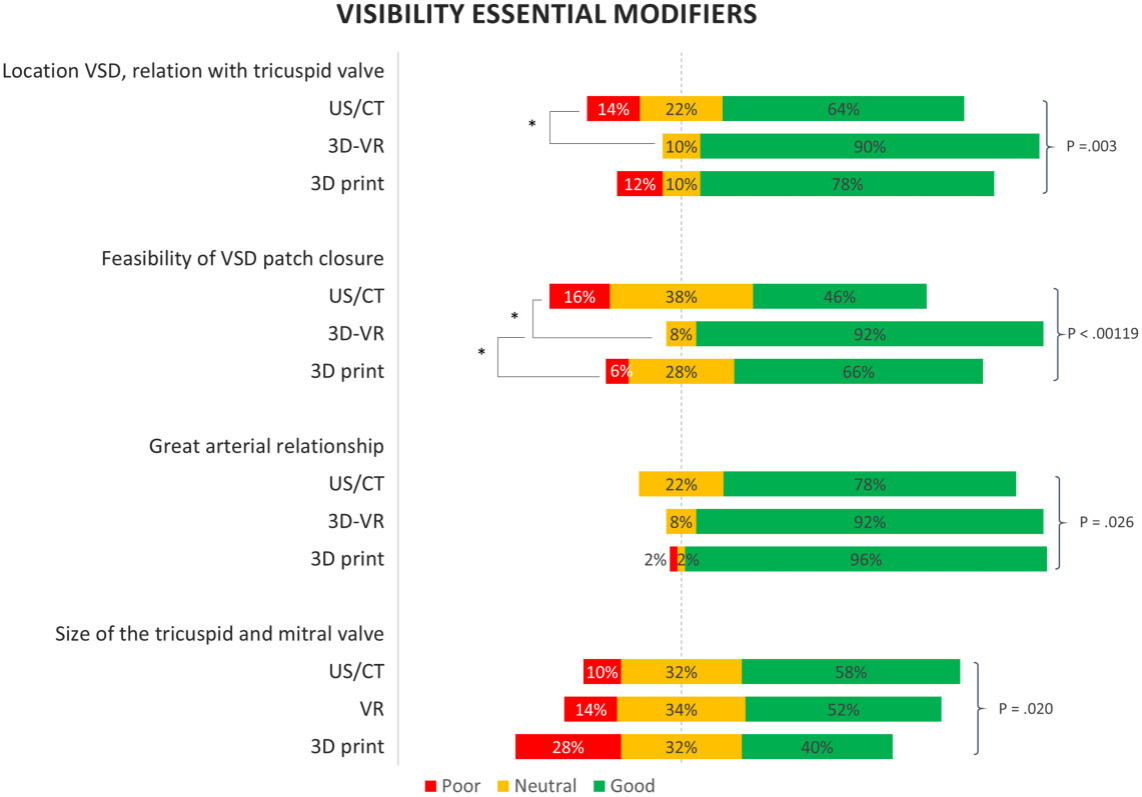
Visibility of the most important essential modifiers for surgical planning. Based on 2D ultrasound and computed tomography images (US/CT), 3D-Virtual Reality (3D-VR) and 3D printing. The *P* values are based on the likelihood ratio test, based on the linear mixed-effect model. *P<0.00111 of the *post hoc* analysis between the groups.

The visibility of the 4 essential modifiers that are most important for DORV surgery are visualized in Fig. 5. The spatial relationships were generally visible best using both 3D modalities. The location of the ventricular septum defect (good visible: US/CT 64%, VR 90%, 3D print 78%, *P *= 0.003) and the feasibility of ventricular septum defect patch closure were best visible on the VR 3D reconstructions (good visible: US/CT 46%, VR 92%, 3D print 66%, *P* < 0.00119). The great arterial relationship was visible best on the 3D printed models (good visible: US/CT 78%, VR 92%, 3D print 96%, *P* = 0.026). Contrarily, the sizes of the mitral and tricuspid valves were best visible using US/CT (good visible: US/CT 58%, VR 52%, 3D print 40%, *P* = 0.020). Systemic and pulmonary venous connections were similarly visible on all 3 modalities (Supplementary Material, Fig. S1).

### Surgical plan

**Figure 6:**
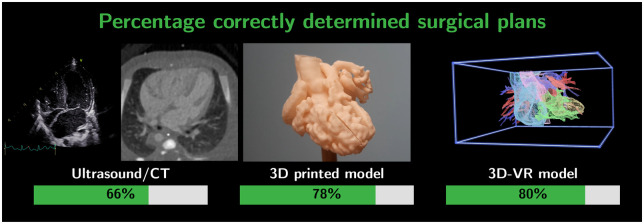
Number of surgical plans corresponding to the actual performed procedure, based on ultrasound and CT images (conventional 2D imaging), 3D printed models, and the 3D-VR models (left to right).

The median time for surgical planning was 14 min (IQR: 8–20) for conventional imaging, 7 min (IQR: 4–11) for VR and 4 min (IQR: 2.5–6) for 3D printing. The proposed surgical plans were compared with the performed surgical procedures. After VR visualization, most surgical plans were corresponding to the performed procedure (correct estimated surgical plans based on: CT/US: 66%, VR: 80% and 3D printing: 78%, Fig. 6). For example, discrepancy between the surgical plans occurred in cases where participants answered that a Nikaidoh or Rastelli would have been performed where an intraventricular tunnel was performed during surgery. Furthermore, we compared the results regarding surgical plan between the 2 specialisms, both surgeons and cardiologists proposed a strategy that was in accordance with the performed surgery in 75% (Supplementary Material, Table S1). The participants were least sure formulating a surgical plan based on the conventional imaging compared with both 3D visualization methods [certainty scores; CT/US: 7.0 (IQR: 5.3–8), VR: 8.0 (IQR: 7.8–9.0) and 3D print: 8.0 (IQR: 7.0–9.0), *P*<0.00119].

### Usefulness, satisfaction and ease of use

Nine participants filled in the questionnaire on the usefulness, satisfaction, ease of use and ease of learning of the 2 3D visualization methods (Supplementary Material, Figs S2–S5). According to the participants, both 3D visualization methods were useful for surgical planning (3D printing: 72%, 3D-VR: 100%) and helped with the understanding of the complex anatomy of the DORV patients (3D printing: 63%, 3D-VR: 100%). Furthermore, most participants prefer to use both 3D visualization methods as additional surgical planning tools (3D printing: 72%, 3D-VR: 100%), but 81% of the participants disagreed to use these methods as a replacement of the gold standard. 3D printed models were easier to use than the 3D-VR reconstructions (3D print: 82%, 3D-VR: 54%), but both methods were applicable without instructions, based on most of the participants’ opinions (3D printing: 90%, 3D-VR: 82%).

## DISCUSSION

In this pilot study, we investigated the additional value of both 3D-VR and 3D printing for surgical planning of complex DORV patients. The use of both additional 3D modalities was found very useful for congenital cardiac surgeons and cardiologists. Spatial relationships needed for surgical planning were better visible in the 3D visualization, and the surgical plans based on the 3D visualizations were more often corresponding to the actual performed surgical procedure. Moreover, the specialists were more certain of their proposed surgical plans after assessing the 3D methods. Based on these observations, we demonstrated the importance of 3D visualization for surgical planning of these complex cases.

For all DORV patients included in this study, both 3D visualizations were found useful and gave more insight in the anatomy and possible surgical reparations than the conventional 2D imaging. The participants’ mental interpretation of the anatomy was confirmed or adjusted through 3D visualization. The proposed surgical plans were more corresponding to the performed surgical procedures, based on 3D printing and 3D-VR. This ability to indicate the right surgical plan prior to the procedure, based on preoperative imaging could diminish the amount of exploration needed intraoperatively. This could save intraoperative time, avoid unnecessary incisions, and associated comorbidities, and therefore may result in better outcomes [3–5].

In VR, the intracardiac structures could be assessed more completely, because both the model and the cutting plane can be moved and visualized in all arbitrary angles easily, using the VR controllers and virtual cutting planes [20]. Furthermore, navigation and orientation were easy in the 3D-VR models because of the colour coding of cardiac segmentations. Thus, 3D-VR models are highly interactive to view extra- and intracardiac structures, dimensions and relationships [21]. For the 3D printed models, the cutting planes were fixed by the designer of the model and therefore some intracardiac structures were only partially accessible. Moreover, due to limited printing resolution, in some models, small structures such as coronary arteries could not be printed, which could have affected the results regarding 3D printing. The 3D prints provided true sized and tactile information, resembling the actual cardiac size intraoperatively. Although, in VR, the 3D models could be scaled up, compared to the scaling by the loupe glasses of the surgeons [22, 23].

Functional and quantitative data, such as the size and straddling of cardiac valves, were better visible on conventional imaging. This is because 3D visualization is limited by the underlying image quality. Because of the limited temporal and spatial resolution of CT, the highly dynamic valves and small structures are often invisible on 2D cross-sectional CT images, making it also impossible to visualize them in physical or virtual 3D reconstructions [24]. This underlines the importance of multimodality imaging. In the future, it could be useful to combine functional and spatial information from multiple imaging modalities, so all necessary information can be obtained in a glance presented preferably in 3 dimensions.

Despite most of the participants being early adopters or early majority with regards to new technology (Table 1), most of them did not prefer to replace the gold standard with the 3D visualizations, but would use it as an addition to the gold standard. Currently, the 3D print and 3D-VR are both based on the CT scans and are thus designed as an addition to the CT and US imaging.

### Limitations

A limitation of this pilot study is the limited number of study subjects (*n* = 5) as well as the small (*n* = 12) and heterogeneous (surgeons and paediatric cardiologists) number of participants. The initial dataset consisted of 11 DORV patients, of which only 5 patients had a preoperative CT scan with adequate spatial and contrast resolution for VR visualization. Because of the variation in the types of DORV patients and surgical approaches, the patient group was suitable for this pilot study, comparing conventional 2D imaging, 3D printing and 3D-VR in terms of visualization. Even though we acknowledge the fact that 3D printing is a technique that has been presented as a possible preoperative surgical planning tool for this challenging patient population, we aimed to compare the added value of 3D printing with the more recently introduced immersive VR-technology that potentially has other benefits than 3D printing. Moreover, we included a relatively small number of participants. Despite the multicentre study design, including both cardiothoracic surgeons as cardiologists, still only 75% of the participants were able to rate all patient cases due to busy working schedules, as it took approximately 25 min for them to analyse each patient case. We also do acknowledge the limitation that we asked cardiologists to propose a ‘surgical repair’ strategy. However, this is resembling the heart team multidisciplinary meetings, in which cardiologists and surgeons discuss the treatment options and surgical strategy for these complex cases. In this study, we demonstrated that also cardiologists are able to estimate the surgical plan correctly. Considering the positive findings regarding both 3D modalities in this retrospective pilot study, the next step is a prospectively designed clinical study including more study subjects and participants. Hence, objective evidence could be gathered on the value of the 3D visualization methods for surgical planning of DORV patients, investigating outcomes such as cardiopulmonary bypass time, intraoperative exploration time, intraoperative changes of plan, residual defects, complications and mortality [15, 25]. However, operative time and patient outcomes are depending on many other patient- and surgeon-related factors, so it remains difficult to study this in this heterogeneous patient population. Currently, we only compared 2D vs 3D printing and 3D-VR. Additionally, direct volume rendering techniques (not requiring segmentation of structures) on a computer monitor could be added as an extra comparison.

In the end, the operative strategy is chosen by the attending surgeon, not necessarily precluding a possibly different other surgical choice. Furthermore, some of the participating surgeons operated on the included DORV patient cases, so they could have recognized these patients’ imaging. This could have resulted in a higher percentage of correctly determined surgical plans. However, this will be higher for all groups (CT, VR and 3D printing), since they assessed all imaging data of the patient cases.

Due to the heterogeneity of DORV patients, various scanning protocols are used within different centres, which may have contributed to the exclusion of 6 cases. It is important to investigate the optimal scanning parameters and use a standardized protocol to obtain comparable and good quality CT images. Furthermore, both statistical tests used (Wilcoxon signed rank and linear mixed-effect model tests) were actually suboptimal for this dataset. The Wilcoxon signed rank test is only able to test 2 groups, instead of the 3 groups (US/CT, VR, 3D print) compared in this study. Moreover, the likelihood ratio test of the linear mixed-effect model is originally described for continuous data, as the Likert-scale results were ordinal data. This could have resulted in more statistical significant results, since more value is assigned to the ordinal data.

### Future perspectives

Since most CHDs are very variable with highly individual anatomy, it can be beneficial to perform 3D surgical planning for various other CHDs besides DORV patients (for example, pulmonary stenosis with collateral arteries, transposition of the great arteries or tracheomalacia). At last, the possibilities of extended reality beyond virtual reality, such as augmented or mixed reality, could be explored. Augmented or mixed reality could be used to create an overlay of the surgical planning on the intraoperative view and serve as intraoperative navigation [21]. In this way, the virtual 3D models are valuable not only preoperatively, but also intraoperatively.

## CONCLUSION

Concluding, this study showed that both 3D printing and 3D-VR have additional value for cardiac surgeons and paediatric cardiologists over 2D imaging, because of better visualization of spatial relationships. As a result, the proposed surgical plans based on the 3D visualizations matched the actual performed surgery to a greater extent. However, a larger and prospective cohort of patients is needed in future research, to quantify results such as patient outcomes, operative time, complications and survival objectively.

## Supplementary Material

ivad072_Supplementary_Data

## Data Availability

The data underlying this article cannot be shared publicly due to privacy of the patients and participants. The data will be shared on reasonable request to the corresponding author. **Jette J. Peek:** Conceptualization; Data curation; Formal analysis; Investigation; Methodology; Resources; Software; Visualization; Writing—original draft). **Wouter Bakhuis:** Supervision; Validation; Writing—review & editing. **Amir H. Sadeghi:** Conceptualization; Methodology; Software; Supervision; Writing—review & editing. **Kevin M. Veen:** Data curation; Formal analysis; Software; Validation; Writing—review & editing. **Arno A.W. Roest:** Conceptualization; Methodology; Resources; Supervision; Writing—review & editing. **Nico Bruining:** Conceptualization; Methodology; Supervision; Writing—review & editing. **Theo van Walsum:** Conceptualization; Methodology; Supervision; Writing—review & editing. **Mark G. Hazekamp:** Conceptualization; Methodology; Resources; Supervision; Writing—review & editing. **Ad J.J.C. Bogers:** Conceptualization; Methodology; Resources; Supervision; Writing—review & editing. Interdisciplinary CardioVascular and Thoracic Surgery thanks Katarzyna Januszewska, Julie Cleuziou and the other, anonymous reviewer(s) for their contribution to the peer review process of this article.

## ABBREVIATIONS

2D: 2 dimensional
3D: 3 dimensional
CHD: Congenital heart disease
CT: Computed tomography
DORV: Double outlet right ventricle
IQR: Interquartile range
US: Ultrasound
VR: Virtual reality
