# Supplementary material for: Optimized preoperative planning of double outlet right ventricle patients by 3D printing and virtual reality: a pilot study
Source: Interdiscip Cardiovasc Thorac Surg. 2023 May 18;37(2):ivad072. doi: 10.1093/icvts/ivad072 (PMC10481772; doi:10.1093/icvts/ivad072)
Supplement: ivad072_Supplementary_Data [file ivad072_Supplementary_Data.zip › Supplementary_File_S1_to_S2.docx]

Supplementary File S1 to S2

Supplementary File S1 - Checklist Essential modifiers

Are the following essential structures and relationships well assessable using the visualization technology?

| Visibility of intra cardiac structures & relationships | Poor | Neutral | Good |
| --- | --- | --- | --- |
| 1. Size of the VSD |  |  |  |
| 1. Location of the VSD from the RV and relation with the Tricuspid valve ostium |  |  |  |
| 1. Size of the tricuspid/mitral valve ostium and straddling of the tricuspid/mitral-chordae |  |  |  |
| 1. Orientation of outlet septum versus VSD |  |  |  |
| 1. Muscular infundibulum presence and extent |  |  |  |
| 1. Great arterial relationships |  |  |  |
| 1. Outflow tract obstruction, presence or absence |  |  |  |
| 1. Feasibility to make a tunnel through the VSD, plane to attach a VSD patch |  |  |  |
| 1. Size of both ventricles |  |  |  |
| 1. Appearance of other associated abnormalities    - - Coronary arterial origins and distribution      - Anomalous systemic venous connection      - Anomalous pulmonary venous connection |  |  |  |

1. Based on this visualization technology, what would be the preferred surgical approach?

- Biventricular repair
- Univentricular Fontan palliation

1. In case of **biventricular** repair, write down the **surgical plan**, based on this visualization technology (eg. Intra-ventricular baffle with a conduit from RV to PA, closing VSD with a patch with or without arterial switch, REV procedure, Nikaidoh, Rastellli )?
2. How certain are you of the surgical plan on a scale from 1 (completely unsure) to 10 (100% sure)

| 1 | 2 | 3 | 4 | 5 | 6 | 7 | 8 | 9 | 10 |
| --- | --- | --- | --- | --- | --- | --- | --- | --- | --- |

After assessing all 3 visualization technologies (CT, VR and 3D print) of this patient

1. Please rank the 3 technologies for surgical planning of choice (for this patient case) best (1) to least (3):

CT – VR – 3D print

1.

2.

3.

*Yim D, Dragulescu A, Ide H, et al. Essential Modifiers of Double Outlet Right Ventricle: Revisit with Endocardial Surface Images and 3-Dimensional Print Models. Circ Cardiovasc Imaging. 2018;11(3). doi:10.1161/circimaging.117.006891*

Supplementary File S2 - Participant Characteristics & Usefulness, Satisfaction and Ease of use questionnaire

**Participant Characteristics**

Profession: _____________________

Work experience in congenital cardiac surgery or paediatric cardiology (years): ­­____

1. Do you have experience with gaming consoles (e.g. computer gaming, xbox, playstation)?

- I have never used a gaming console
- I have used a gaming console a few times before
-
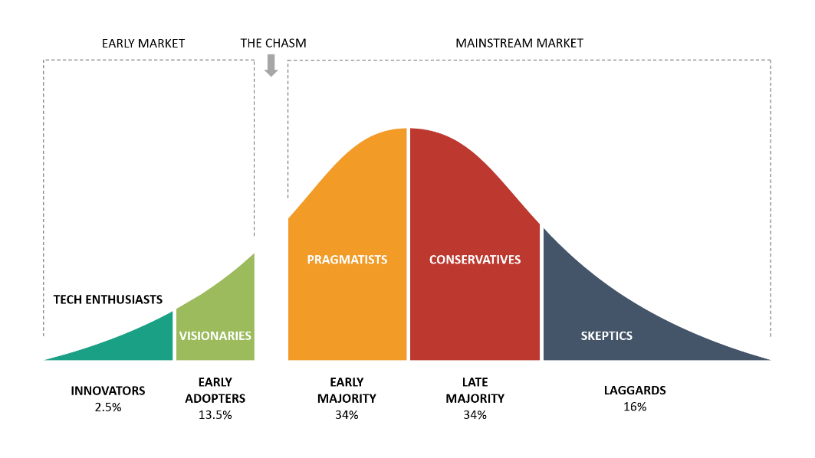
I am gaming on a regular basis (at least once a month)

1. With respect to using new technologies in general, to what segment do you belong of the Innovation Adoption Curve of Rogers?
   - Innovators
   - Early adopters
   - Early majority
   - Late majority
   - Laggards

Rogers, E.M. (1995). Diffusion of Innovations. 4th ed., New York: The Free Press

1. How often do you use Virtual Reality hardware/software (e.g. VR gaming, simulations, consoles, entertainment etc.)?

- I have never had a VR experience until today
- I have used VR a few times before
- I am experienced and use VR on a regular basis (at least once a month)
- I am an VR expert (have a VR console and applications myself)

1. Do you have experience with using physical 3D printed models for surgical planning?

- I have never used 3D printed models
- I have used 3D printed models a few times before
- I use 3D printed models on a regular basis

1. Do you think **Virtual Reality** 3D models will become a standard visualization method in preoperative planning for complex congenital heart surgery

| Fully disagree | Disagree | Neutral | Agree | Fully agree |
| --- | --- | --- | --- | --- |

1. Do you think **3D printed models** will become a standard visualization method in preoperative planning for complex congenital heart surgery

| Fully disagree | Disagree | Neutral | Agree | Fully agree |
| --- | --- | --- | --- | --- |

| **USE questionnaire 3D printing** | | 1. Fully disagree | 2. Disagree | 3. Neutral | 4. Agree | 5. Fully agree | N/A |
| --- | --- | --- | --- | --- | --- | --- | --- |
|  | **Usefullness** |  |  |  |  |  |  |
| 1 | The 3D printed model aids to be more effective |  |  |  |  |  |  |
| 2 | The 3D printed model is useful |  |  |  |  |  |  |
| 3 | The 3D printed model gives me more control on the surgical planning |  |  |  |  |  |  |
| 4 | The 3D printed model makes surgical planning easier to get done |  |  |  |  |  |  |
| 5 | The 3D printed model saves me time when I use it |  |  |  |  |  |  |
| 6 | The 3D printed model meets my needs |  |  |  |  |  |  |
|  | **Ease of use** |  |  |  |  |  |  |
| 1 | The 3D printed model is easy to use |  |  |  |  |  |  |
| 2 | The 3D printed model is user friendly |  |  |  |  |  |  |
| 3 | It requires the fewest steps to accomplish what I want to do with the 3D printed model |  |  |  |  |  |  |
| 4 | After initial training, using the 3D printed model is effortless |  |  |  |  |  |  |
| 5 | After initial training, I can use the 3D printed model without instructions |  |  |  |  |  |  |
| 6 | I can recover from mistakes quickly and easily |  |  |  |  |  |  |
| 7 | When the model is ready to use, I can use the 3D printed model successfully every time |  |  |  |  |  |  |
| 8 | Interaction with the 3D printed model is of additional value for understanding the complex DORV anatomy |  |  |  |  |  |  |

|  | **Ease of learning** |  |  |  |  |  |  |
| --- | --- | --- | --- | --- | --- | --- | --- |
| 1 | I learned to work with the 3D printed model quickly |  |  |  |  |  |  |
| 2 | I easily remember how to use the 3D printed model |  |  |  |  |  |  |
| 3 | It is easy to learn to use a 3D printed model |  |  |  |  |  |  |
| 4 | I quickly became skillful with the 3D printed model |  |  |  |  |  |  |
|  | **Satisfaction** |  |  |  |  |  |  |
| 1 | I am satisfied with the 3D printed model |  |  |  |  |  |  |
| 2 | I would recommend using this 3D printed model to other colleagues |  |  |  |  |  |  |
| 3 | The 3D printed model is fun to use |  |  |  |  |  |  |
| 4 | I would prefer 3D printed models instead of the golden standard 2D imaging (echo, CT) |  |  |  |  |  |  |
| 5 | I would prefer 3D printed models additionally to the golden standard 2D imaging (echo, CT) |  |  |  |  |  |  |
| 6 | The threshold to use a 3D printed model is still too high for me (for example given the required effort and time) |  |  |  |  |  |  |
| 7 | I think 3D printed models will become a standard visualization method in preoperative planning for complex congenital heart surgery |  |  |  |  |  |  |

*Lund A. Measuring Usability with the USE Questionnaire. Usability User Exp Newsl STC Usability SIG. 2001;8.*

**Advantages / disadvantages**

Write down the advantages and disadvantages of the 3D printed model for surgical planning of DORV patients, rank them in order of importance, from most important (1) to least important (3).

**Advantages:**

1.

2.

3.

**Disadvantages:**

1.

2.

3.

| **USE questionnaire 3D VR reconstruction** | | 1. Fully disagree | 2. Disagree | 3. Neutral | 4. Agree | 5. Fully agree | N/A |
| --- | --- | --- | --- | --- | --- | --- | --- |
|  | **Usefullness** |  |  |  |  |  |  |
| 1 | The 3D VR reconstruction aids to be more effective |  |  |  |  |  |  |
| 2 | The 3D VR reconstruction is useful |  |  |  |  |  |  |
| 3 | The 3D VR reconstruction gives me more control on the surgical planning |  |  |  |  |  |  |
| 4 | The 3D VR reconstruction makes surgical planning easier to get done |  |  |  |  |  |  |
| 5 | The 3D VR reconstruction saves me time when I use it |  |  |  |  |  |  |
| 6 | The 3D VR reconstruction meets my needs |  |  |  |  |  |  |
|  | **Ease of use** |  |  |  |  |  |  |
| 1 | The 3D VR reconstruction is easy to use |  |  |  |  |  |  |
| 2 | The 3D VR reconstruction is user friendly |  |  |  |  |  |  |
| 3 | It requires the fewest steps to accomplish what I want to do with the 3D VR reconstruction |  |  |  |  |  |  |
| 4 | After initial training, using the 3D VR reconstruction is effortless |  |  |  |  |  |  |
| 5 | After initial training, I can use the 3D VR reconstruction without instructions |  |  |  |  |  |  |
| 6 | I can recover from mistakes quickly and easily |  |  |  |  |  |  |
| 7 | When the model is ready to use, I can use the 3D VR reconstruction successfully every time |  |  |  |  |  |  |
| 8 | Interaction with the 3D VR reconstruction is of additional value for understanding the complex DORV anatomy |  |  |  |  |  |  |

|  | **Ease of learning** |  |  |  |  |  |  |
| --- | --- | --- | --- | --- | --- | --- | --- |
| 1 | I learned to work with the 3D VR reconstruction quickly |  |  |  |  |  |  |
| 2 | I easily remember how to use the 3D VR reconstruction |  |  |  |  |  |  |
| 3 | It is easy to learn to use a 3D VR reconstruction |  |  |  |  |  |  |
| 4 | I quickly became skillful with the 3D VR reconstruction |  |  |  |  |  |  |
|  | **Satisfaction** |  |  |  |  |  |  |
| 1 | I am satisfied with the 3D VR reconstruction |  |  |  |  |  |  |
| 2 | I would recommend using this 3D VR reconstruction to other colleagues |  |  |  |  |  |  |
| 3 | The 3D VR reconstruction is fun to use |  |  |  |  |  |  |
| 4 | I would prefer 3D VR reconstruction instead of the golden standard 2D imaging (echo, CT) |  |  |  |  |  |  |
| 5 | I would prefer 3D VR reconstruction additionally to the golden standard 2D imaging (echo, CT) |  |  |  |  |  |  |
| 6 | The threshold to use a 3D VR reconstruction is still too high for me (for example given the required effort and time) |  |  |  |  |  |  |
| 7 | I think 3D VR reconstruction will become a standard visualization method in preoperative planning for complex congenital heart surgery |  |  |  |  |  |  |

*Lund A. Measuring Usability with the USE Questionnaire. Usability User Exp Newsl STC Usability SIG. 2001;8.*

**Advantages / disadvantages**

Write down the advantages and disadvantages of the 3D VR reconstruction for surgical planning of DORV patients, rank them in order of importance, from most important (1) to least important (3).

**Advantages:**

1.

2.

3.

**Disadvantages:**

1.

2.

3.
