# Supplementary material for: Optimized preoperative planning of double outlet right ventricle patients by 3D printing and virtual reality: a pilot study
Source: Interdiscip Cardiovasc Thorac Surg. 2023 May 18;37(2):ivad072. doi: 10.1093/icvts/ivad072 (PMC10481772; doi:10.1093/icvts/ivad072)
Supplement: ivad072_Supplementary_Data [file ivad072_Supplementary_Data.zip › Supplementary_Figures_S1_to_S5_revised.docx]

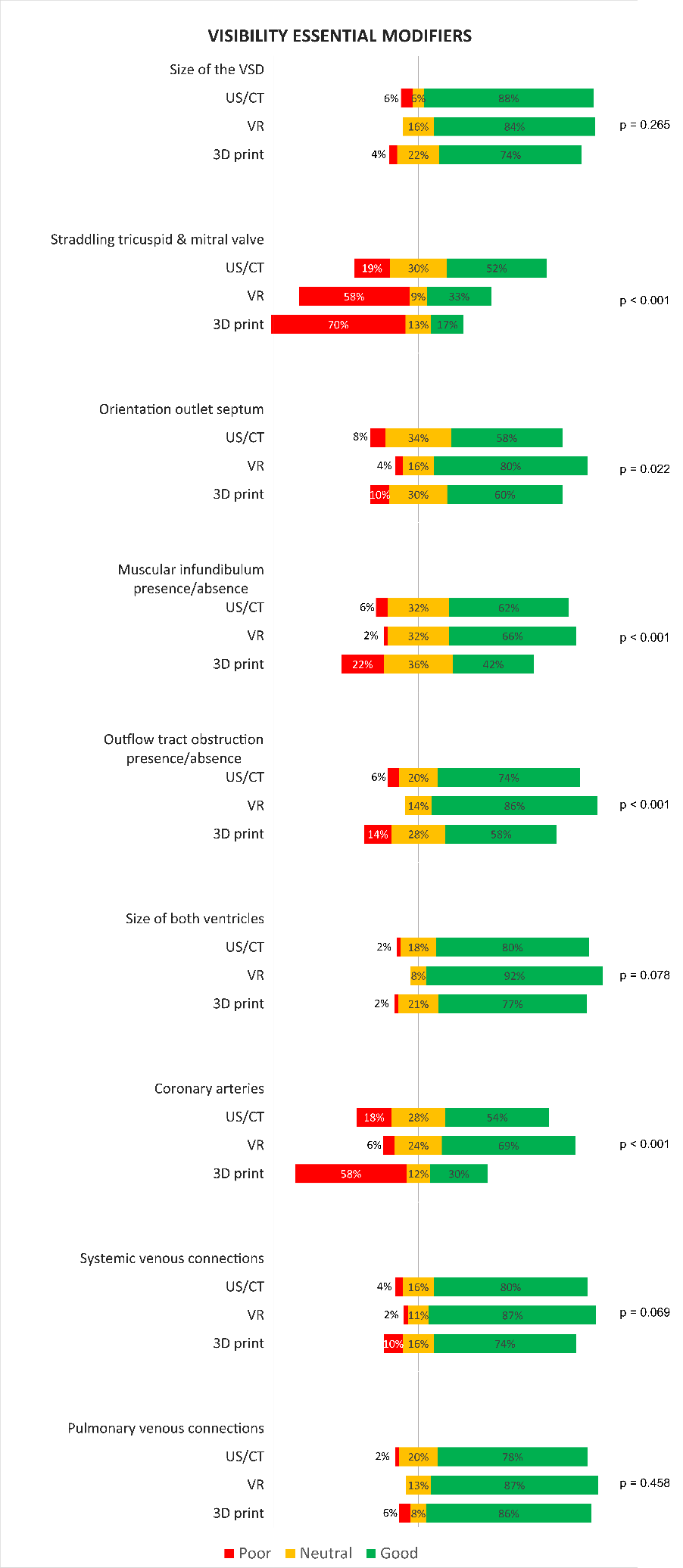
**Supplementary Figures S1 to S5**

**Supplementary Figure S1.** Visibility of Essential modifiers based on ultrasound and CT images (US/CT), 3D printing and Virtual Reality (VR)


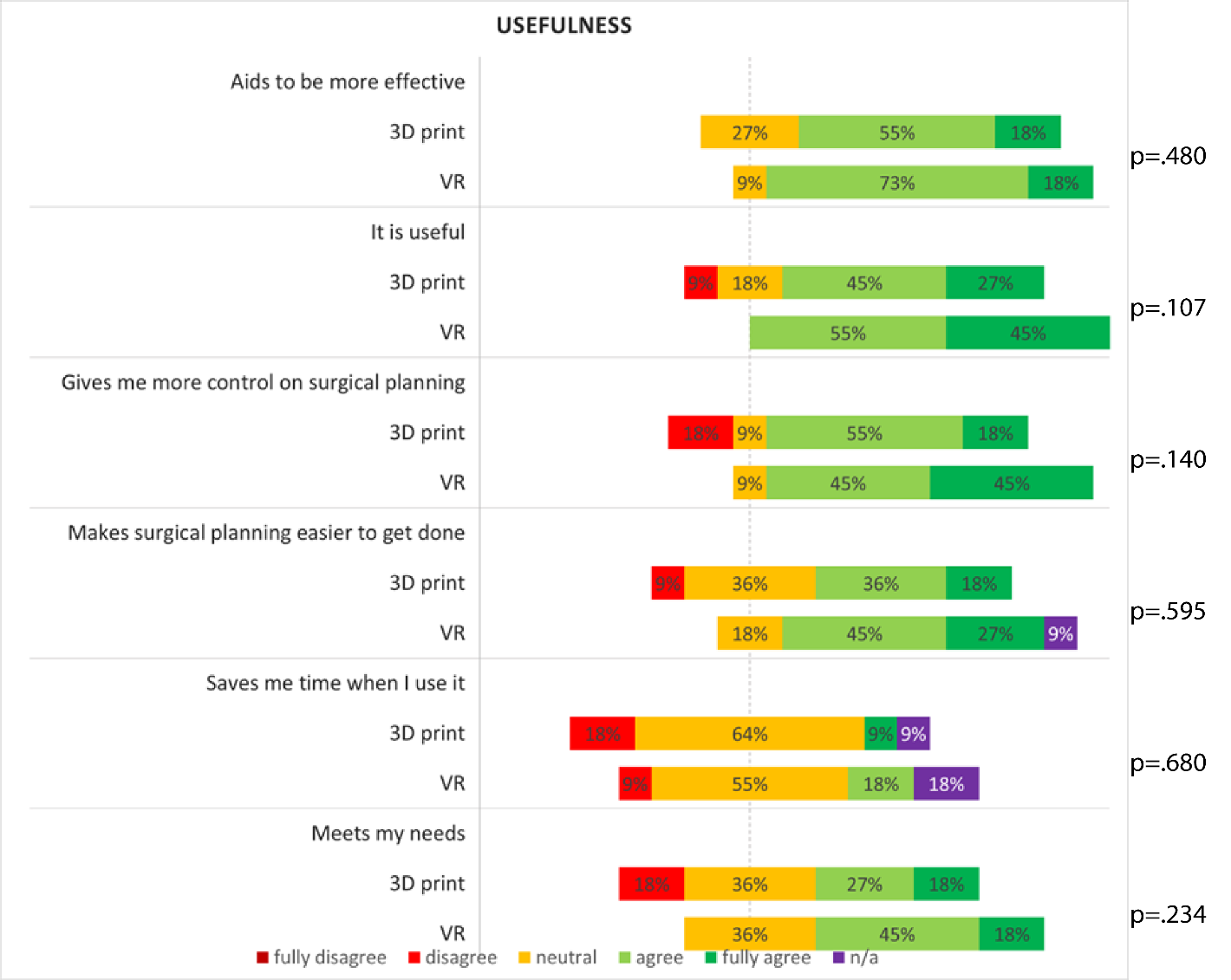


**Supplementary Figure S2.** Representation of the usefulness of 3D printed vs 3D Virtual Reality (VR) models


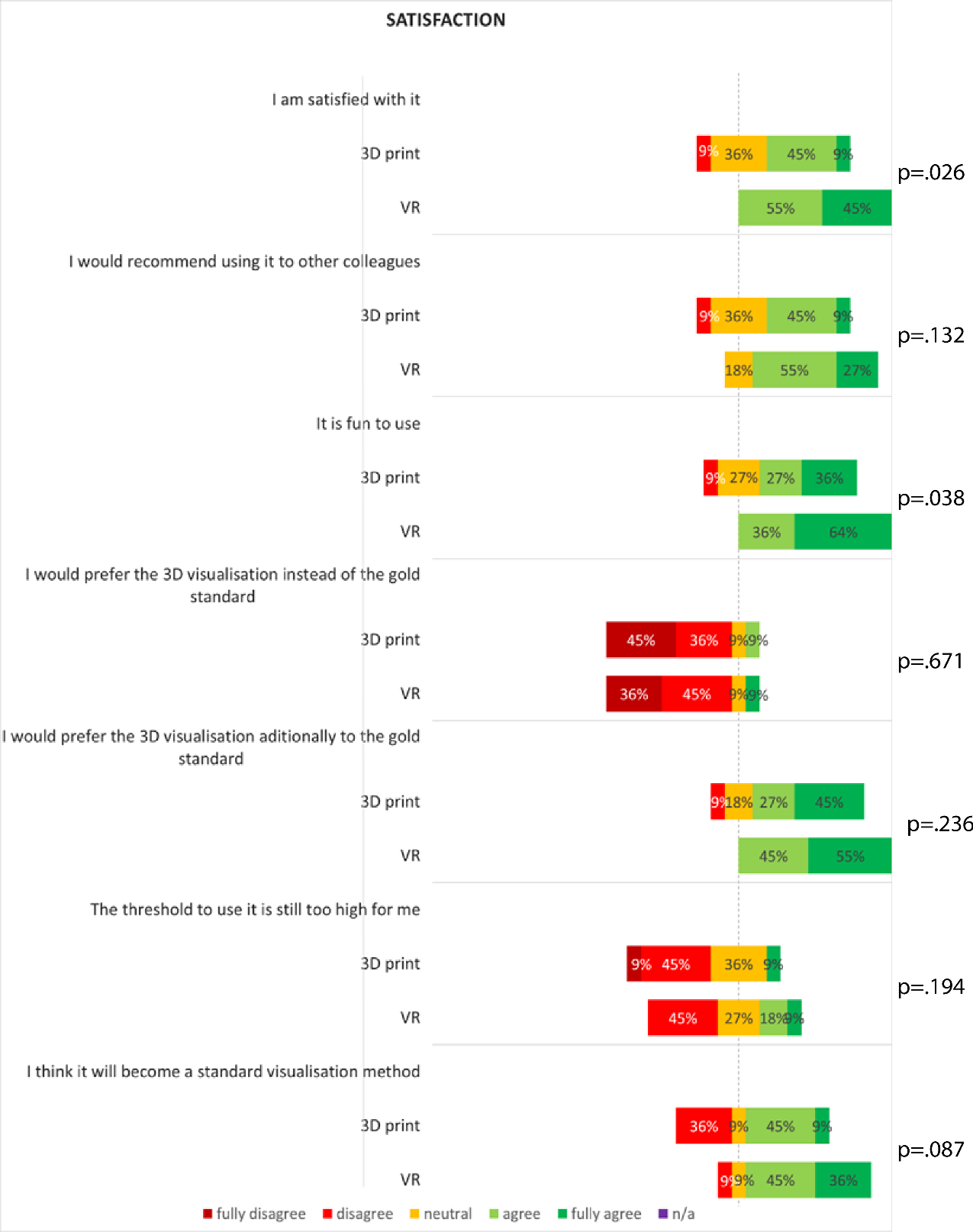


**Supplementary Figure S3.** Representation of the satisfaction of the 3D printed vs the 3D Virtual Reality (VR) models


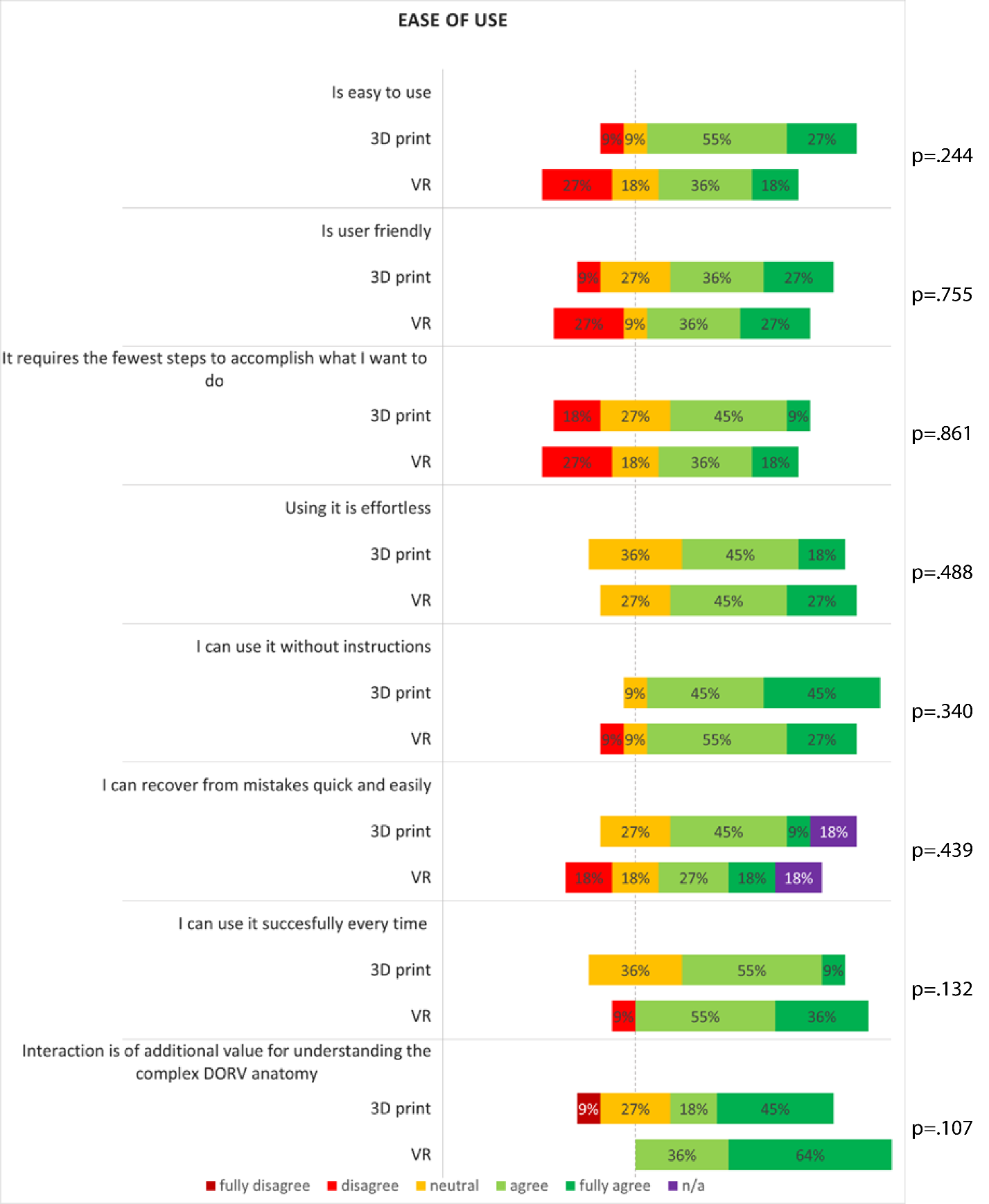


**Supplementary Figure S4.** Representation of the ease of use of the 3D printed vs the 3D Virtual Reality (VR) models


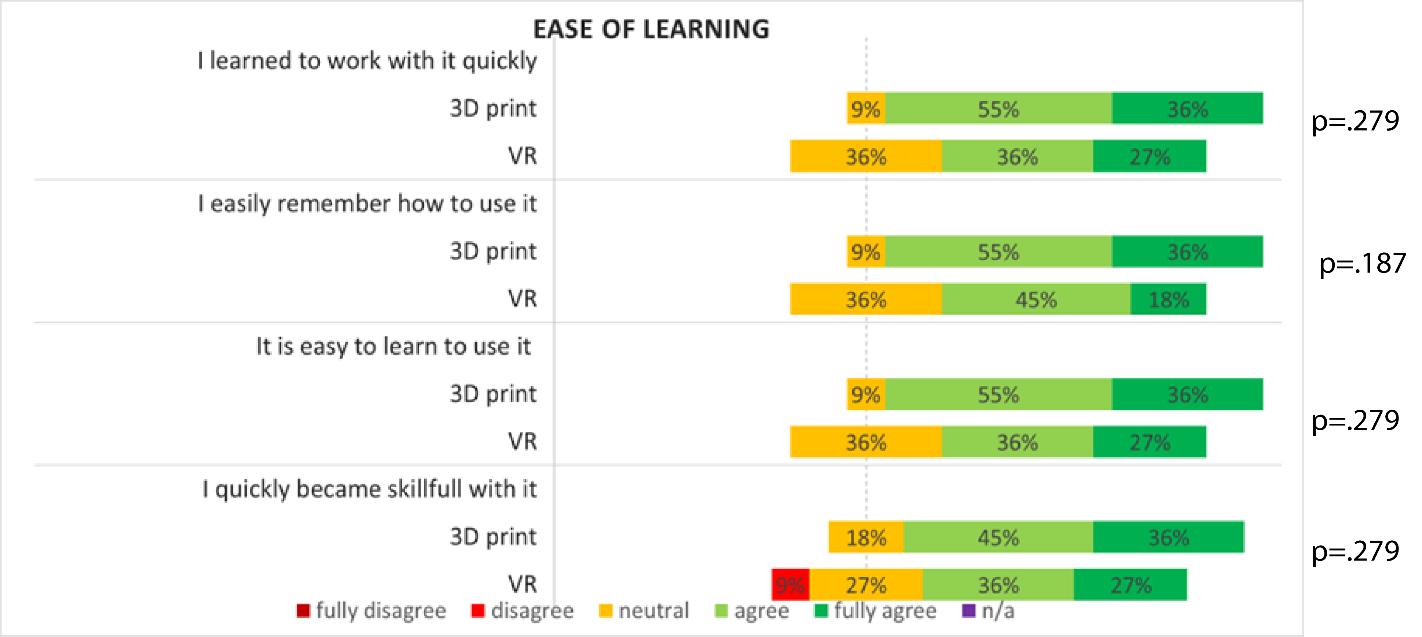


**Supplementary Figure S5.** Representation of the ease of learning of the 3D printed vs the virtual reality 3D models
